# Identifying potential binding sites for complex formation between Tyrosyl-DNA phosphodiesterase 1 and poly [ADP-ribose] polymerase 1

**DOI:** 10.1039/d6cp00941g

**Published:** 2026-05-19

**Authors:** Sophia Wang, Aykut Üren, Purushottam B. Tiwari

**Affiliations:** a Department of Biology, Georgetown University Washington D.C. 20057 USA; b Department of Oncology, Georgetown University Washington D.C. 20057 USA pbt7@georgetown.edu +1(202) 687 3841

## Abstract

DNA topoisomerases manage the supercoiled structure of the genomic DNA through breaking and rejoining DNA strands, which is a key step in many cellular processes. DNA topoisomerase I (TOPI) forms TOPI–DNA cleavage complex (TOPIcc) *via* formation of a transient covalent bond between TOPI and the DNA single strand. Inhibition of TOPI enzymatic activity is a successful approach for treating multiple types of cancer. Tyrosyl-DNA phosphodiesterase 1 (TDP1) helps release of TOPIccs *via* catalysis of TOPI–DNA phosphodiester bond hydrolysis. TDP1 is therefore functionally connected with activity of TOPI. Poly [ADP-ribose] polymerase 1 (PARP1) physically interacts with TDP1 to make TDP1 PARylated and enhances TDP1 recruitment to DNA damage sites, thus playing a functional role in TOPIcc repair. If unrepaired, TOPIcc can lead to single strand breaks, which cause cell death. Slowing down TDP1 activity can increase the efficacy of existing TOPI inhibitors and improve their clinical utility. Repair of TOPIccs can be negatively impacted by blocking the physical interactions between TDP1 and PARP1. Therefore, blocking the TDP1–PARP1 complex formations has the potential to enhance the antitumor activity of existing FDA approved TOP1 inhibitors and reduce their side effects. Towards this aim, we identified binding sites that are crucial for the TDP1–PARP1 complex formation using 700 ns long molecular dynamics (MD) simulations. We identified specific interactions between D115(TDP1) and K940 or K943 (PARP1) as well as R137(TDP1) and E883(PARP1) that might be important for the TDP1–PARP1 complex formation. We validated the complex formation between purified recombinant TDP1 and PARP1 proteins using surface plasmon resonance (SPR). We also used SPR to confirm that peptides corresponding to contact points between TDP1 and PARP1 prevent complex formation. Our findings lead the path for creating novel inhibitors that can prevent TDP1 binding to PARP1 and consequently improve the clinical efficacy of the current TOP1 inhibitors for cancer treatment.

## Abstract 2

PARP1 physically interacts with TDP1 and plays a functional role in repair of the DNA topoisomerase I-DNA cleavage complex.

## Graphical abstract

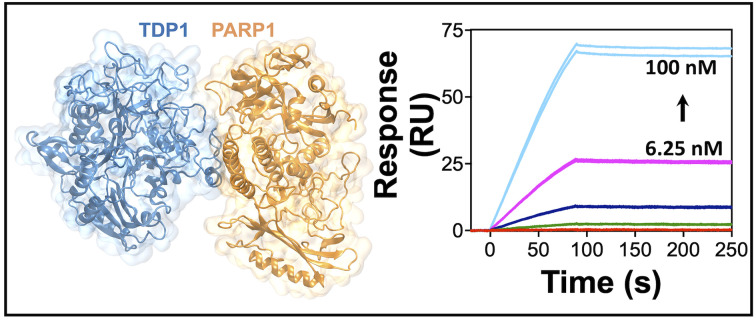

## 1. Introduction

Human DNA topoisomerase I (TOPI) is a validated target enzyme for cancer treatment with multiple FDA approved drugs in the clinic.^1–6^ TOPI establishes a transient covalent bond with the single strand of DNA and forms TOPI–DNA cleavage complex (TOPIcc) as an important step in the DNA repair mechanism.^1,7^ Hydrolysis of the TOPI–DNA phosphodiester bond is catalyzed by tyrosyl-DNA phosphodiesterase 1 (TDP1), which can reverse the TOPI activity.^8,9^ TDP1 thus has an important role in repairing DNA damage caused by TOPIccs that are trapped on DNA strands^10^ through hydrolysis of the phosphodiester bond between TOPI and DNA.^11^ If TOPIccs are not released, this may result in DNA strand break, which results in cell death.^12^ TOPI inhibitors have been used in the clinic for decades^5,6^ but their use still has challenges.^13^ Irinotecan, an FDA approved-TOPI inhibitor and a camptothecin (CPT) derivative, is approved for colorectal cancer treatment;^5,6^ topotecan is approved for treatments of metastatic ovarian cancer, small cell lung cancer (SCLC), and cervical cancer,^6^ and these inhibitors pose dose-limiting toxicities.^6,14–17^

The functional interaction between TDP1 and TOPI plays a critical role in tumor cells^18^ such that effectiveness of TOPI inhibitors are correlated with slower TDP1 activity.^19^ In cells with elevated TDP1 expression, CPT, a TOPI inhibitor, causes less DNA damage.^20^ To enhance its ability to repair damage due to formation of TOPIccs, TDP1 forms a protein–protein complex with poly (ADP-ribose) polymerase 1(PARP1), in which TDP1 becomes PARylated by PARP1.^11,21,22^ The PARylation leads to recruitment of TDP1 to DNA damage sites.^11^ Therefore, physical interaction between TDP1 and PARP1 has functional significance in repair of TOPIccs. Recent as well as past studies have been conducted on combined PARP and TOPI inhibitions utilizing PARP1 inhibitors, with the aim of improving cancer treatments.^23–27^ However, PARP inhibitors pose toxicity risks in cancer treatments.^28–33^ We are also focused on achieving the same outcome without inhibiting PARP1. We believe that preventing PARP1–TDP1 interaction is likely to have fewer side effects compared to inhibiting PARP1 enzymatic activity. Hence, development of novel inhibitors that block formation of TDP1–PARP1 complex may improve the effectiveness of TOPI inhibitors. Proper characterization of the TDP1–PARP1 complex formation and identification of potential binding sites may lead to development of inhibitors to block the TDP1–PARP1 complex formation.

The N-terminal domain (NTD) of TDP1 physically interacts with C-terminal domain (CTD) of PARP.^11^ However, key amino acid residues in these two proteins that are responsible for the formation of the interprotein complex have not been established yet. In this report, we predict binding sites that are involved in the TDP1–PARP1 complex formation. We first computationally predicted the structure of the TDP1–PARP1 complex. We used this approach to characterize multiple protein–protein interactions.^34–37^ We used surface plasmon resonance (SPR)-based technique to experimentally validate the complex formation. SPR is a label-free biophysical technique to study direct binding of biomolecules,^38–41^ and we have successfully used this biophysical technique to investigate protein–protein interactions.^34,35,37,42,43^ Our investigations were then continued with 700 ns long MD simulations of the predicted TDP1–PARP1 complex, similar to MD simulations we have conducted in the past for other protein–protein complexes.^34,36,37,44,45^ MD simulation-based studies are very useful in drug discovery research, including target modeling and interaction of protein with other binding partners.^46–48^ MD simulation-based results together with experimental data enhance accuracy as well as validity of predicted binding sites.^46,48^

Our results suggest that TDP1 binds to PARP1 with a nanomolar binding affinity and the TDP1–PARP1 complex was stable for 700 ns long MD simulations. Our analysis of the 700 ns long MD simulation trajectories revealed key amino acid pairs that might be important for the TDP1–PARP1 complex formation. We also calculated computational binding affinity using the MM/GBSA approach as we did in our most recent publications.^36,37,49^ SPR results further confirmed the involvement of specific residues in the TDP1–PARP1 complex formation. We believe that our detailed characterization of the TDP1–PARP1 complex formation *via* identification of potential binding sites would be a valuable contribution to scientific community conducting research to develop novel inhibitors to improve effectiveness of TOPI inhibitors.

## 2. Materials and methods

### 2.1. Proteins, sensorchips, and reagents

PARP1 (catalog# 11040-H08B, full-length) was purchased from Sino Biological (Houston, TX). TDP1 (catalog# TDP1-7250H, 1-298 aa) was purchased from Creative Biomart (Shirley, NY). Glutathione *S*-transferases (GST, catalog# BR100223), Series S CM5 sensorchips (catalog# 29149603), amine coupling kit (catalog# BR100050), HBS-P+ buffer (catalog# BR100671) were purchased from cytiva (Marlborough, MA). Synthetic peptides (PT-PP1-1 and PT-PP1-2) were purchased from GenScript (Piscataway, NJ).

### 2.2. Preparation and optimization of individual TDP1 and PARP1 structures

Full-length TDP1 was modelled using I-TASSER webserver^50^ and pdb ID 1JY1^51^ as well as TDP1 FASTA sequence (NCBI accession# AAH15474.1^52^). Visual Molecular Dynamics (VMD) software^53^ was used in structural alignment and comparison. Full-Length PARP1 was modelled using modeler and pdb IDs 4DQY,^54^ 2COK,^55^ and 3ODC^56^ as well as PARP1 FASTA sequence (NCBI accession# NP_001609.2^52^). VMD^53^ was used in structural alignment of individual PARP1 domains. Notably, only missing residues in the crystal structures were fulfilled from the predicted structures of both TDP1 and PARP1 *via* structural overlapping with available crystal structures to obtain the proteins in their full-length forms for better docking prediction. The full-length structures of TDP1 and PARP1 were optimized *via* all-atom molecular dynamics (MD) simulation for 100 ns using the NAMD software (version 2.14 or 3.0).^57^

### 2.3. Prediction of the TDP1–PARP1 complex

Coordinates at the end of 100 ns all-atom simulations of individual full-length TDP1 and PARP1 structures were used to predict the TDP1–PARP1 complex using PatchDock web server^58^ and the top 50 predictions from the PatchDock complexes were submitted to FireDock web server for refinement.^59,60^ The top-ranked complex from the 50 refined FireDock results was considered for further analysis. This top-ranked complex was visualized in VMD^53^ and this complex, without PARP1 regions that were far away from the TDP1–PARP1 binding interface, was used as the predicted TDP1–PARP1 complex for further investigations using 700 ns all-atom MD simulations. The removal of the distant PARP1 regions offered a reduced system size with significantly shorter simulation times.

### 2.4. Molecular dynamics (MD) simulations

All-atom MD simulations of the individual TDP1 and PARP1 structures as well as the predicted TDP1–PARP1 complex were conducted, for 700 ns, using the NAMD software (version 2.14 or 3.0)^57^ and CHARMM36m force field^61^ as we conducted in the past.^34,36,37,44,48,49,62^ Briefly, VMD^53^ or CHARMM-GUI webserver^63^ was used to generate simulation input files. The simulation systems in cubic boxes were solvated with TIP3 water and NaCl was added for neutralization. The dimension of cubic boxes for TDP1 only was 100 × 100 × 100 Å^3^ with total number of atoms 95 007. The dimension of cubic boxes for PARP1 only was 170 × 170 × 170 Å^3^ with total number of atoms 472 500. The dimension of cubic boxes for final truncated TDP1–PARP1 complex prepared for production runs was 130 × 130 × 130 Å^3^ with total number of atoms 205 445. The solvated systems were minimized for 10 000 steps and then equilibrated for 100 ps under the NVT ensemble at a time step of 1 fs at 300 K at 1 atm pressure and Langevin dynamics with a damping constant of 1 ps^−1^ with the conjugate gradient and line search algorithm. The production runs were then propagated at a time step of 2 fs under the NPT ensemble for 100 ns for the individual structures during initial optimization and 700 ns for the final predicted complex at 300 K and 1 atm pressure and Langevin dynamics with a damping constant of 1 ps^−1^. Four independent replica runs for the TDP1–PARP1 complex were conducted for better confidence of our results. Root mean square deviation (RMSD) values were used to assess stability of the 700 ns MD simulation trajectories.

### 2.5. Surface plasmon resonance (SPR)

SPR measurements were carried out using a Biacore T200 instrument with CM5 chips at 25 °C. PARP1 was first diluted in 10 mM sodium acetate buffer at pH 5.5 and then immobilized as ligand onto the CM5 chips to levels of ∼2700–3200 RU using amine coupling chemistry. We also immobilized Glutathione *S*-transferases (GST) to levels of ∼700–1400 RU using the same procedure as used for PARP1. Here, GST was used as a control protein (ligand). A reference flow cell (FC) was prepared adjacent to the active FCs, which were used to immobilize PAPRP1 or GST, by activating and deactivating using the same surface chemistry as the active FCs but no ligands were immobilized onto this reference FC. HBS-P (10 mM Hepes pH 7.4, 150 mM NaCl, 0.05% surfactant P20), 10× diluted HBS-P+ in ddH_2_O, was used as the immobilization running buffer (buffer running in background during immobilization). TDP1 at different concentrations in HBS-P supplemented with 0.2% v/v glycerol was injected as analyte over the reference and active (ligand immobilized) surfaces. 100 nM TDP1 only, 400 µM to 25 µM peptides only, and mixtures of 100 nM TDP1 and peptides in HBS-P supplemented with 0.2% v/v glycerol were also injected as analytes during inhibition assays. HBS-P supplemented with 0.2% v/v glycerol was used as running buffer during all analyte-ligand binding experiments. Each concentration of all analytes was injected in duplicate. A flow rate of 30 µL min^−1^ was maintained during injection of all analytes. A 20 s pulse of glycine at pH 2.0 was injected for surface regeneration. All SPR sensorgrams recorded for further analysis were double referenced (blank, which is the buffer only response and reference, which is response corresponding to the reference FC subtracted). SPR sensorgrams recorded for mixtures of TDP1 and peptides were further processed by subtracting peptide only sensorgrams from the sensorgrams obtained for the TDP1-peptide mixtures.

### 2.6. Data analysis

MD simulation trajectories were analyzed using visual molecular dynamics (VMD).^53^ Interfacial contact residues and salt bridges were analyzed using a 3.5 Å distance cutoff. Hydrogen bonds were predicted using a 3.5 Å distance cutoff and with a 30° angle cutoff. Binding free energies were estimated using the MM/GBSA approach and NAMD as done in our previous publications.^36,37,49^ GraphPad Prism was used to plot data. Carma^64^ was used to measure radius of gyration (*R*_g_).

## 3. Results and discussion

### 3.1. Preparation of TDP1 and PARP1 structures and prediction of the TDP1–PARP1 complex

**Fig. 1:**
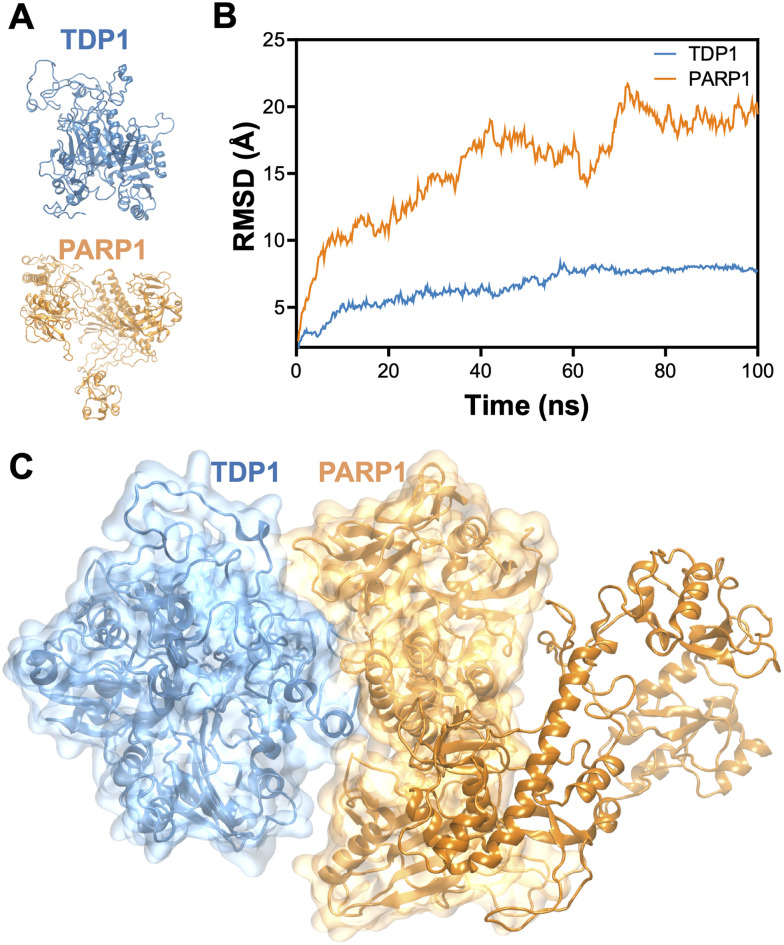
Prediction of individual structures and the TDP1–PARP1 complex. (A) Optimized TDP1 and PARP1 full-length structures. (B) RMSD measurements showing the stability of full-length individual TDP1 and PARP1 structures. The structural stabilities of both structures were observed after ∼75 ns. (C) Predicted TDP1–PARP1 complex using optimized TDP1 and PARP1 structures at 100 ns.

We predicted full-length TDP1 and PARP1 structures as outlined in the MATERIALS AND METHODS section above. Since full length-length protein structures were predicted by modeling missing residues as well as structural overlapping with the available crystal structures, we first optimized the individual full-length TDP1 and PARP1 structures *via* 100 ns MD simulations. MD simulations allowed structural flexibility and reorganization as needed. Fig. 1A depicts TDP1 and PARP1 full-length structures at the end of 100 ns MD simulations.

We monitored structural integrity of the individual structures *via* root mean square deviation (RMSD) measurements. As shown in Fig. 1B, both structures were stable after ∼75 ns. Therefore, we used the optimized structures of both TDP1 and PARP1 at the end of 100 ns to predict the TDP1–PARP1 complex. In Fig. 1B, much bigger RMSDs were observed for PARP1. Protein with multiple domains connected with flexible linkers can cause such higher RMSD values^65–67^ and PARP1 is a multidomain protein connected with flexible linkers.^68^Fig. 1C is the predicted full-length TDP1–PARP1 complex using the procedures as mentioned in the MATERIALS AND METHODS section. We analyzed the top-predicted complex structure and found that TDP1-N-terminal domain (NTD) was complexed with PARP1-C-terminal domain (CTD). Since this observation for NTD(TDP1)-CTD(PARP1) interactions for the complex formation agreed well with a prior experimental prediction,^11^ we decided to proceed with this complex. We believed that simulation outcomes are more reliable with a choice of an initial structure that is close to experimental predictions, whenever available. There are many residues in PARP1 that are far away from the TDP1–PARP1 binding interface. We removed these PARP1 residues (residue IDs 1 to 495 represented in dark orange color in Fig. 1C) and prepared the final TDP1–PARP1 complex for subsequent MD simulation studies for 700 ns. This truncation was very helpful in greatly reducing simulation time for multiple replica runs.^37^

### 3.2. Confirmation of TDP1–PARP1 direct binding using surface plasmon resonance (SPR)

**Fig. 2:**
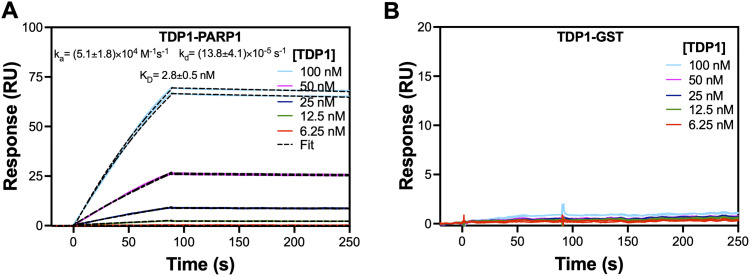
Confirmation of TDP1–PARP1 direct binding using SPR. (A) Representative concentration-dependent binding of TDP1 to immobilized PARP1 on a CM5 chip surface. Colored lines are experimental data and dashed lines are fit to a 1 : 1 binding model. The association rate constant (*k*_a_), dissociation rate constant (*k*_d_), and equilibrium dissociation constant (*K*_D_, affinity) were determined from three independent set of analyte injections. (B) SPR sensorgrams showing lack of direct binding of TDP1 to immobilized control protein, GST, on the CM5 chip surface.

We conducted SPR-based biophysical experiments to confirm TDP1–PARP1 direct binding. As shown in Fig. 2A, we obtained clear direct binding of TDP1 to PARP1. PARP1 was immobilized onto the CM5 chip surface. Different colored lines are experimental data showing concentration-dependent binding of TDP1 to immobilized PARP1. We injected each concentration of TDP1 in duplicate to monitor technical reproducibility. When we fitted the experimental data (dashed lines) to a 1 : 1 binding model, we obtained a *K*_D_ (affinity) value of 2.8 ± 0.5 nM for the TDP1–PARP1 complex formation with slower dissociation rate constant. The *K*_D_ values are presented as the mean ± s.d. from three independent set of analyte (TDP1) injections. To the best of our knowledge, this is the first experimental quantification of affinity for the formation of this functionally important TDP1–PARP1 complex. We also conducted control experiments by immobilizing GST as a negative control protein onto the CM5 chip surface using the same procedures as adopted for PARP1 and then injected TDP1 solutions with the same range of concentrations. This control experiment did not show any binding of TDP1 to GST (Fig. 2B). This confirms that the TDP1–PARP1 binding that we obtained was a specific interaction.

### 3.3. Molecular dynamics simulations of the TDP1–PARP1 complex and complex stability

**Fig. 3:**
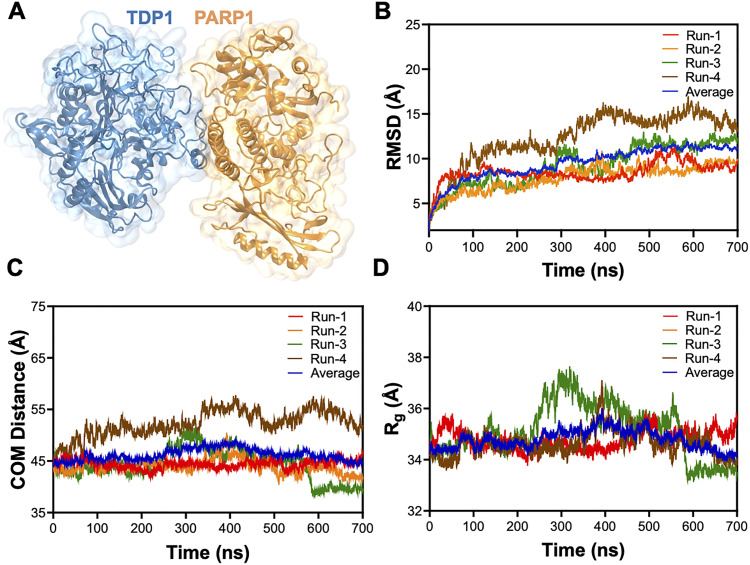
TDP1–PARP1 complex structure. (A) Representative TDP1–PARP1 complex structure at the end of 700 ns all-atom MD simulations. (B) RMSD (C) center of mass (COM) distance between TDP1 and PARP1, and (D) radius of gyration (*R*_g_) measurements for four different 700 ns all-atom MD simulations of the TDP1–PARP1 complex. Blue trace represents the average values from four different replica runs.

We conducted 700 ns all-atom MD simulations of the predicted TDP1–PARP1 complex. Our 700 ns all-atom MD simulations showed that the TDP1–PARP1 complex was stable for 700 ns of the simulation time. Fig. 3A is a representative TDP1–PARP1 complex structure at the end of 700 ns MD simulation. The representative protein coordinates in pdb format for this structure are provided in the SI (Section S1) and a representative simulation movie in the Section S2 (SI). Proteins are flexible biomolecules and the conformational dynamics correspond to structure as well as function of proteins.^69^ Computational investigations, including MD simulations, are useful in investigating how the conformational flexibility in a protein helps accommodate its binding partner and the stability of the complex of the protein with its binding partner.^70,71^ In the past, we have successfully utilized MD simulations to investigate interaction of proteins with their binding partners.^34,36,37,44,45,48,49,62^

Fig. 3B shows RMSD measurements for four different independent runs for the TDP1–PARP1 complex and the blue line represents the average RMSDs from the four different runs. RMSD values are helpful in evaluating better convergence and stable conformations of the simulated systems.^72–74^ Higher RMSD values in Fig. 3B might be due to the large number of PARP1 domains connected with flexible linkers as explained above. Fig. 3B shows that RMSD values for all complex structures are stable after ∼500 ns of the all-atom MD simulations, despite big changes due to initial structural reorganizations as expected. We also analyzed center of mass (COM) distance between TDAP and PARP1 in the complex and performed radius of gyration (*R*_g_) measurements, and results are presented in Fig. 3C and 3D, respectively. Fairly stable COM distance and *R*_g_ measurements also did not show significant structural destabilization. These results together with RMSD measurements altogether predict stability of the TDP1–PARP1 complex structures.^72,73,75^

### 3.4. Analysis of TDP1–PARP1 binding interface and identification of interfacial contacts

**Fig. 4:**
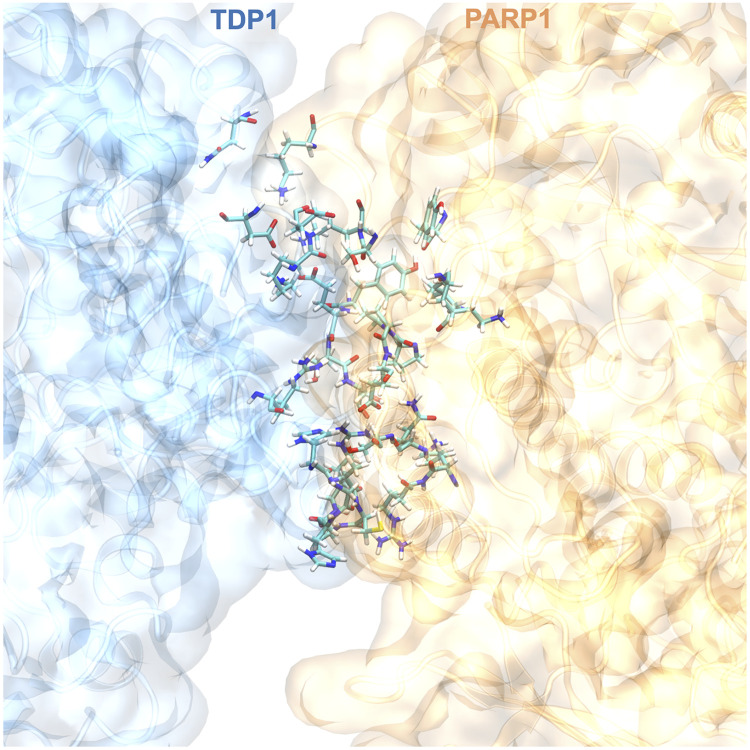
Interfacial contacts. Amino acid residues in licorice representation that establish interfacial contacts across the TDP1–PARP1 binding interface.

This work is aimed at investigating potential TDP1–PARP1 binding sites that can be exploited to develop novel drugs to enhance efficacy of current inhibitors targeting TOPI. We analyzed the 700 ns long all-atom MD simulation trajectories of the TDP1–PARP1 complex to identify potential residues that likely establish interfacial contacts between the two proteins across the binding interface. We considered the presence of the same residue pairs formed in at least three out of four MD simulation runs. These residues are shown in licorice representation in Fig. 4. Table 1 shows the list of these identified residues.

**Table 1:** List of amino acid residues in both TDP1 and PARP1 that establish interfacial contacts across the TDP1–PARP1 binding interface

TDP1	PARP1
ASN67	LYS943
LYS112, GLU113, ASP115	LYS940
ARG126	PRO882, GLU883
HSD130	GLN707, SER711, GLU883
GLY131	GLN707, SER711
ALA132	ARG704
ALA134	GLN707
CYS135	LYS703, ARG704, GLN707
HSD136	ARG704
ARG137	GLU883
TYR145	ILE879, LYS893, SER939, LYS940, TYR992
GLU146, THR147	PRO882
SER148	PRO881, PRO882

It is useful to focus on clusters of contacting residues across the binding interface as a potential binding pocket for development of novel inhibitors to block the complex formation between these two proteins. The contact region comprises of residues on surfaces and hence defines binding between the two proteins. These contact residues are responsible for the specificity and binding strength between the proteins.^76^ Therefore, these interfacial contact residues across the TDP1–PARP1 binding interface contribute to overall affinity between these two proteins. We also quantified the binding affinity *via* determination of binding free energy for the TDP1–PARP1 complex formation using the same MM/GBSA approach that we utilized in our prior publications^36,37,49^ and we obtained a favorable binding free energy of −44.3 ± 12.2 kcal mol^−1^ (mean ± s.d. from four different runs). To the best of our knowledge, this is the first computational quantification of affinity for the formation of this functionally important TDP1–PARP1 complex.

### 3.5. Determination of hydrogen bonding and salt bridges responsible for TDP1–PARP1 complex formation

**Fig. 5:**
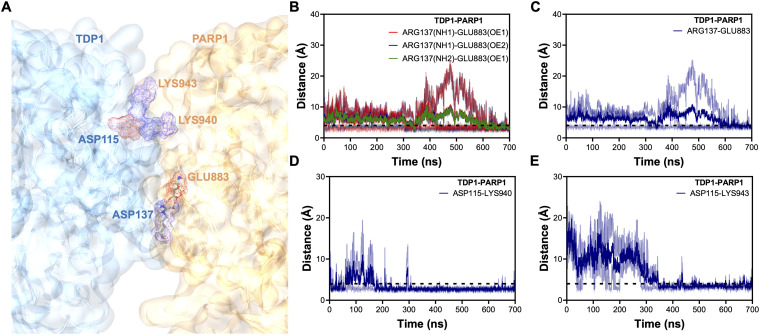
Hydrogen bonding and salt bridges between TDP1 and PARP1. (A) Location of amino acid residues that were predicted to establish hydrogen bonding and salt bridge in three dimensional (3D) complex structure. Distance–time plots for (B) predicted hydrogen bonding and (C), (D), and (E) for salt bridges. The atom types that are responsible for hydrogen bonding are shown inside parentheses in Fig. 5B for each predicted amino acid residue. The same light-colored data in Fig. 5B–E correspond to distance–time measurements from different runs with the average values shown in dark colors.

We analyzed 700 ns all-atom MD simulation trajectories to identify key amino acid residues that form specific non-covalent interactions. We considered the same pairs of interactions that are present in at least three out of four replica runs. We found that the residue pair involving ARG137(TDP1) and GLU883(PARP1) was responsible for hydrogen bonding. Fig. 5A shows the location of these amino acid residues across the TDP1–PARP1 binding interface. Fig. 5B shows the distance–time curve for the atoms that establish the hydrogen bonding. The dashed line corresponds to the 3.5 Å cutoff that we used to analyze the hydrogen bonds. The average values from the different runs are shown in darker colors while the individual values shown in the same lighter colors. There was a large fluctuation in distances between atoms in one of the runs that caused the average distance–time curve for the hydrogen bonds in Fig. 5B to be slightly above the cutoff line until ∼600 ns, while the binding distance in other runs was below the threshold. The same residue pair shown in Fig. 5B was also predicted to establish the salt bridge and the residue–residue distance for this salt bridge is shown in Fig. 5C. The same reasoning for fluctuation of hydrogen bond distance–time curve as explained above applies for the average distance–time curve for this salt bridge to be slightly above the 3.5 Å cutoff until ∼600 ns. We also observed that ASP115 in TDP1 formed salt bridges with LYS940 or LYS943 in PARP1. ASP115 (TDP1) was identified in three runs but the ASP115(TDP1)-LYS940(PARP1) or ASP115(TDP1)-LYS943(PARP1) in only two runs. We analyzed the location of LYS940 or LYS943 in PARP1 in the three dimensional (3D) complex structure and as shown in Fig. 5A, contact regions of these two PARP1 residues are in close proximity that might alternatively or combinedly establish salt bridges with ASP115(TDP1). The distance–time graphs for these two salt bridges are shown in Fig. 5D and E.

### 3.6. Experimental confirmation of involvement of specific residues in the TDP1–PARP1 complex formation

**Fig. 6:**
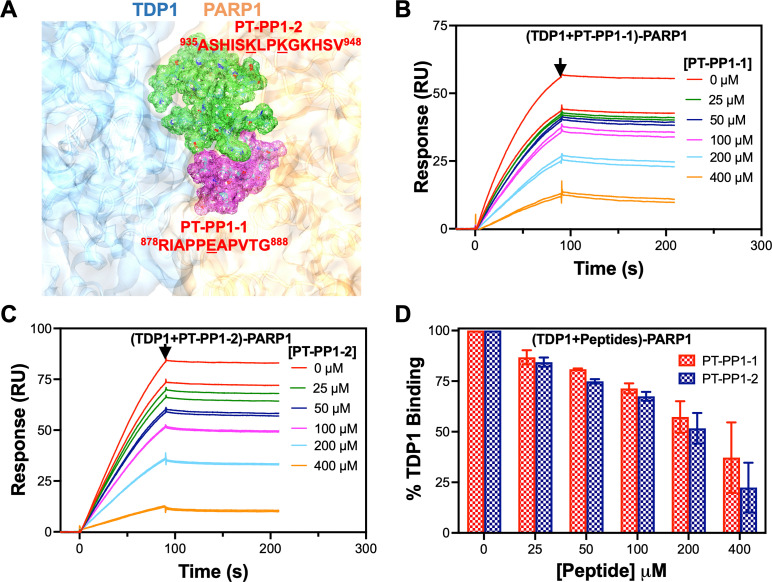
Inhibition of the TDP1–PARP1 complex formation in the absence and presence of synthetic peptides. (A) Location of amino acid residues, used to synthesize two peptides (PT-PP1-1 and PT-PP1-2), in three dimensional (3D) complex structure. SPR sensorgrams showing concentration dependent inhibition of binding of 100 nM TDP1 to immobilized PARP1 in the absence and presence of (B) PT-PP1-1 and (C) PT-PP1-2. (D) Response values at the end of association, represented by the arrows in (B) and (C), presented as percentage bindings of 100 nM TDP1 to immobilized PARP1 in the absence and presence of synthetic peptides. SPR response values obtained for 100 nM TDP1 binding in the absence of either peptide were considered as 100% binding. Heights of bar graphs represent average and error bars are s.d. of the response values obtained from two independent set of analyte injections.

As shown in Fig. 5, three amino acid residues E883, K940, and K943 in PARP1 form specific bonds (hydrogen bonds and/or salt bridges). We designed two peptides PT-PP1-1 and PT-PP1-2 and used these synthesized peptides to experimentally confirm the involvement of these three specific amino acids. It is to be noted that PT-PP1-1 includes E883 and PT-PP1-2 includes K940 and K943 in PARP1. These three PARP1 residues, E883, K940, and K943 are well within the clusters of PARP1 interacting residues as shown in Fig. 4. We designed two peptides instead of a single peptide since E883 is spaced far from K940 and K943, and that would make a single peptide too long. Fig. 6A shows the locations of these two peptides in the 3D complex structure, which are positioned close to each other. Fig. 6A also includes amino acid sequences of these two peptides together with the corresponding range in the PARP1 sequence.

We injected 100 nM TDP1 only and mixtures of 100 nM TDP1 with various concentrations of peptides in HBS-P supplemented with 0.2% glycerol over the PARP1 immobilized surface. As shown in Fig. 6B, binding amplitude (represented by an arrow at the end of association phase) of SPR sensorgrams gradually decreases as concentration of PT-PP1-1 increases. The same trend was observed for PT-PP1-2 as shown in Fig. 6C. We presented the response values at the end of association phase (represented by arrows in Fig. 6B and C) as the percentage bindings of 100 nM TDP1 in the absence and presence of peptides as shown in Fig. 6D. We set the 100 nM TDP1 response value in the absence of either peptide as 100% binding. We believe that both peptides bind to TDP1 in solution, as expected based on results shown in Fig. 6, which showed impaired binding of TDP1 to immobilized PARP1 on the sensor surface, resulting in concentration dependent inhibition of TDP1–PARP1 complex formation in the presence of either peptide. The inhibition efficiency of PT-PP1-2 seemed to be slightly higher than that of PT-PP1-1 and this might be due to presence of two lysines in this peptide that form specific bonding with TDP1 as compared to a single glutamic acid in PT-PP1-1. These results experimentally confirm involvement of the computationally predicted amino acid pairs establishing specific bonds between TDP1 and PARP1. These results further suggest that, while all other amino acids that establish interfacial contact between TDP1 and PARP1 contribute to overall affinity, the amino acid residues that establish specific hydrogen bonds and salt bridges might be important for the complex formation and stabilization.

Altogether, our work has identified potential binding sites responsible for formation and stabilization of the TDP1–PARP1 complex. In the absence of crystal structure of this functionally important complex, our investigation opens further avenues in this research field, including our ongoing work on targeting the TDP1–PARP1 complex to explore novel inhibitors that improve efficacy of TOPI inhibitors.

## 4. Conclusions

TDP1–PARP1 physical interaction has a crucial functional role in TOPIcc repair. Repair of TOPIccs can be negatively impacted by blocking physical interactions between TDP1 and PARP1. Identification of potential TDP1–PARP1 binding sites can lead to development of new inhibitors that block the TDP1–PARP1 complex formation and these novel inhibitors can be used to enhance effectiveness of existing TOPI inhibitors in clinics. In this study, we attempt to identify the potential TDP1–PARP1 binding sites that establish the interprotein complexes. To the best of our knowledge, for the first time, we have determined the key amino acid residues and experimentally as well was computationally quantified the binding affinity value for the TDP1–PARP1 complex formation. In the absence of crystal structure of the functionally important TDP1–PARP1 complex, we believe that our study is helpful in developing novel inhibitors to enhance effectiveness of existing TOPI inhibitors to treat cancers.

## Author contributions

P. B. T. conceived, designed, and supervised the project. S. W. and P. B. T. performed data acquisition. S. W., A. Ü, and P. B. T. analyzed data. S. W. and P. B. T. wrote the manuscript. A. Ü. contributed to manuscript editing.

## Conflicts of interest

The authors declare no competing interests.

## Supplementary Material

CP-028-D6CP00941G-s001

CP-028-D6CP00941G-s002

CP-028-D6CP00941G-s003

CP-028-D6CP00941G-s004

CP-028-D6CP00941G-s005

## Data Availability

A representative PDB files at the end of 700 ns all-atom MD simulations, topology and parameter files, configuration files, and other files including coordinate (.pdb) and structure (.psf) files are provided as the supporting files. Corresponding files for the CHARMM36m force field that were used in this study are available on the MacKerell Lab webpage (https://mackerell.umaryland.edu/charmm_ff.shtml) or on the CHARMM-GUI webpage (https://www.charmm-gui.org/). NAMD and VMD software can be downloaded from the developer's webpage (https://www.ks.uiuc.edu/Development/). CARMA can be downloaded from the Glykos Lab webpage (https://utopia.duth.gr/glykos/Carma.html). Biacore T200 evaluation software version 3.2.1 comes with the Biacore T200 instrument and can be purchased from Cytiva (https://www.cytivalifesciences.com/). GraphPad Prism can be purchased from the GraphPad website (https://www.graphpad.com/). Supplementary information: Protein coordinates in TDP1–PARP1 complex. Simulation movie of the TDP1–PARP1 complex. See DOI: https://doi.org/10.1039/d6cp00941g.
