# Supplementary material for: Identifying potential binding sites for complex formation between Tyrosyl-DNA phosphodiesterase 1 and poly [ADP-ribose] polymerase 1
Source: Phys Chem Chem Phys. 2026 May 19;28(23):14017–27. doi: 10.1039/d6cp00941g (PMC13203358; doi:10.1039/d6cp00941g)
Supplement: CP-028-D6CP00941G-s003 [file CP-028-D6CP00941G-s003.pdf]

Supplementary Information (SI) for Physical Chemistry Chemical Physics.
This journal is © the Owner Societies 2026

Supporting Information

Identifying potential binding sites for complex formation between Tyrosyl-DNA phosphodiesterase 1 and poly [ADP-ribose] polymerase 1

Sophia Wang[1], Aykut Üren[2], Purushottam B. Tiwari[2*]

[1]Department of Biology, Georgetown University, Washington, D.C. 20057, USA

[2]Department of Oncology, Georgetown University, Washington, D.C. 20057, USA

*Corresponding Author

P. B. Tiwari

Email: pbt7@georgetown.edu

Tel: +1(202) 687 3841

S1. Protein coordinates in TDP1-PARP1 complex

A representative file for protein coordinates in the TDP1-PARP1 complex (main text) is presented as a separate supplemental file with name TDP1_PARP1_complex.pdb. In this pdb file, segname PROA corresponds to PARP1 and segname PROB corresponds to TDP1. Sample topology and parameter files, configuration files, and other files including coordinate (.pdb) and structure (.psf) files are provided as separate supporting files (Sample_topology_parameter_files.zip and Sample_configuration_restrain_coordinate_structure_files.zip).

S2. Simulation movie of the TDP1-PARP1 complex

A representative simulation movie for entire 700 ns all-atom MD simulation is uploaded as a separate supplemental file with name TDP1_PARP1_simulation_Movie.mov. In the simulation movie, the light-blue colored protein represents TDP1 and the orange-colored protein represents PARP1. The residues in licorice representation across the interface are the residues in both TDP1 and PARP1 that established interfacial contacts. The lower structures in surface representation with dark-blue and red represent amino acid residues that were predicted to form hydrogen bonding. The same lower residue pairs together with the upper structures in surface representation were predicted to form salt bridges. The contact residues, hydrogen bonding, and salt bridges are also highlighted in Figures 4 and 5, respectively, in the main text.
